# RESULTS FROM RESEARCH ON A HEALTH-COACHED WALKING PROGRAM IN FAMILY CAREGIVERS OF PERSONS WITH DEMENTIA

**DOI:** 10.1093/geroni/igz038.1064

**Published:** 2019-11-08

**Authors:** Jane Flanagan, Kathryn Post, Rebecca Hill

**Affiliations:** 1 Boston College, Chestnut Hill, Massachusetts, United States; 2 Massachusetts General Hospital, Boston, Massachusetts, United States

## Abstract

Caregivers of persons with dementia demonstrate increased levels of stress, anxiety and depression, placing them at increased risk for poor health-related outcomes. Walking is widely supported as a cost-effective, accessible exercise and way to maintain physical fitness and mitigate stress levels. There is a critical gap in addressing health promotion strategies in caregivers. The specific aims of this health-coaching (HC) walking study using wireless pedometers in family caregivers of persons with dementia were to: 1) establish the feasibility of HC and wireless pedometer use, 2) examine outcomes of well-being, stress and activity level and 3) understand the experience of participation. This 2-group comparative study used a repeated measure design and mixed methods approach. We enrolled 27 females and 5 male caregivers (n=32), μ age 57 years: with both 16 in the control in the intervention arm. Pre-post measures of general health (body mass index [BMI], blood pressure, heart rate, cognition, well-being, stress and perceived activity level) were obtained from caregivers at baseline and again at 12 weeks. Results indicate that those who received HC had a statistically significant improvement in BMI (p = .01). There were no other statistically significant improvements in outcomes in either group. Qualitative findings suggest that participants reported many stresses that made self-care challenging. In summary, this was a feasible intervention that resulted in improved BMI in the HC group. Work is needed to understand the long-term impact of this outcome. Further exploration of other health-promoting interventions that may be beneficial for this population is essential.

